# Dissimilar *Trypanosoma cruzi* genotype-specific serological profile assessed by Chagas-Flow ATE IgG1 upon benznidazole etiological treatment of chronic Chagas disease

**DOI:** 10.1371/journal.pntd.0012487

**Published:** 2024-09-13

**Authors:** Glaucia Diniz Alessio, Carolina Malheiros Araújo Silvestrini, Silvana Maria Elói-Santos, Eliane Dias Gontijo, Policarpo Ademar Sales Júnior, Danielle Marchetti Vitelli-Avelar, Renato Sathler-Avelar, Ana Paula Barbosa Wendling, Andréa Teixeira-Carvalho, Marta de Lana, Olindo Assis Martins-Filho

**Affiliations:** 1 Grupo Integrado de Pesquisas em Biomarcadores, Instituto René Rachou (FIOCRUZ-Minas), Belo Horizonte, Brazil; 2 Faculdade de Medicina, Universidade Federal de Minas Gerais (UFMG), Belo Horizonte, Brazil; 3 Instituto Aggeu Magalhães—Fiocruz Pernambuco, Recife, Brazil; 4 Laboratório de Doença de Chagas, Núcleo de Pesquisas em Ciências Biológicas (NUPEB), Instituto de Ciências Exatas e Biológicas (ICEB), Universidade Federal de Ouro Preto (UFOP), Ouro Preto, Brazil; Universidad de la Republica Uruguay, URUGUAY

## Abstract

The present study aimed to verify the impact of etiological treatment on the genotype-specific serological diagnosis of chronic Chagas disease patients (CH), using the Chagas-Flow ATE IgG1 methodology. For this purpose, a total of 92 serum samples from CH, categorized as Not Treated (NT, n = 32) and Benznidazole-Treated (Bz-T, n = 60), were tested at Study Baseline and 5^Years^ Follow-up. At Study Baseline, all patients have the diagnosis of Chagas disease confirmed by Chagas-Flow ATE IgG1, using the set of attributes (“antigen/serum dilution/cut-off”; “EVI/250/30%”). The genotype-specific serodiagnosis at Study Baseline demonstrated that 96% of patients (44/46) presented a serological profile compatible with TcII genotype infection. At 5^Years^ Follow-up monitoring, NT and Bz-T presented no changes in anti-EVI IgG1 reactivity. However, significant differences were detected in the genotype-specific IgG1 reactivity for Bz-T. The most outstanding shift comprised the anti-amastigote TcVI/(AVI), anti-amastigote TcII/(AII) and anti-epimastigote TcVI/(EVI) reactivities. Regardless no changes in the genotype-specific serology of NT (TcI = 6%; TcII = 94%), distinct *T*. *cruzi* genotype-specific sero-classification was detected for Bz-T samples at 5^Years^ Follow-up (TcII = 100%) as compared to Baseline (TcII = 97%; TcVI = 3%). The anti-trypomastigote TcI/(TI) was the attribute accountable for the change in genotype-specific sero-classification. In conclusion, our findings of dissimilar *T*. *cruzi* genotype-specific serology upon Bz-treatment re-emphasize the relevance of accomplishing the genotype-specific serodiagnosis during clinical pos-therapeutic management of chronic Chagas disease patients.

## Author summary

Chagas disease is still a serious public health problem worldwide. Currently, only two drugs are available for the treatment of patients infected by *T*. *cruzi*: Benzonidazole and Nifurtimox. The efficacy of these compounds may differ depending on the phase of the disease when the treatment is established and also impacted by the *T*. *cruzi* genotypes that cause the infection. Thus, differences in therapeutic efficacy can be observed between geographic areas due to the distinct distribution of “*Discrete Typing Unitys*” (DTUs) of *T*. *cruzi*. Besides all these matters related to the etiological treatment, additional concerns regarding the laboratorial methods available for post-therapeutic monitoring of Chagas disease represent a challenge during clinical management. Amongst the innovative serological approaches, proposed for diagnosis and post-therapeutic monitoring of Chagas disease, the Chagas-Flow ATE IgG1 has been presented as an outstanding methodology, applicable for universal and genotype-specific serology. In the present study, the Chagas-Flow ATE IgG1 methodology was used for post-therapeutic monitoring of chronic Chagas disease patients, aiming at identifying changes in *T*. *cruzi* genotype-specific serological profile upon Benznidazole etiological treatment. This approach is relevant to provide novel insights to support the relevance of accomplishing the genotype-specific serodiagnosis during clinical pos-therapeutic management of chronic Chagas disease patients.

## Introduction

Chagas disease, caused by the parasite *Trypanosoma cruzi*, is a serious public health problem, affecting 6–7 million people around the world with 10,000 deaths every year, mainly in Latin America [1]. Regardless of the high number of Chagas disease patients eligible to receive etiological treatment, currently, only two drugs, available since the beginning of the 70s, have been used for therapeutic intervention: Nifurtimox (Lampit) and Benznidazole (Rochagan and Radanil) [2]. Moreover, the efficacy of these compounds may differ depending on the phase of the disease when the treatment is established and also impacted by the *T*. *cruzi* genotypes that causes the infection [3–6]. The major concern regarding the therapeutic success is the low efficacy rates (2–40%) reported during chronic infection [2,4,6–11]. Additionally, as far as the *T*. *cruzi* genetic variability, differences in the therapeutic effectiveness can be observed amongst geographic areas due to the distinct distribution of *T*. *cruzi* “*Discrete Typing Unitys*” (DTUs) [5,12–17]. Moreover, the occurrence of mixed infections may also impact the treatment response of chronic infection [18].

Besides all these matters related to the etiological treatment, additional concerns regarding the laboratorial methods available for post-therapeutic monitoring of Chagas disease represent a challenge during clinical post-therapeutic management. The persistent positive results of conventional serological methods, the low performance of parasitological/molecular tests and the long-term follow-up requirement (>20 years) remain the most obstacle for post-therapeutic cure assessment of patients with chronic Chagas disease [8,10,19,20]. In this sense, although the conventional serological methods (Hemagglutination, Indirect Immunofluorescence and Enzyme-Linked Immunosorbent Assay) have been universally proposed for diagnosis and post-therapeutic monitoring of the Chagas disease, their performance can differ depending on the target antigen used [14,20–24]. Likewise, it is also possible that the infecting *T*. *cruzi* DTU may impact the timing of seroreversion [20].

The use of non-conventional serological methods has been pointed out as an alternative to reduce the timespan required for post-therapeutic monitoring of chronic Chagas disease [25–27]. Amongst the innovative serological approaches, proposed for diagnosis and post-therapeutic monitoring of Chagas disease, the Chagas-Flow ATE IgG1 has been presented as an outstanding methodology, applicable for universal and genotype-specific serology [24,27–29]. The Chagas-Flow ATE IgG1 is a single competitive flow cytometry platform for simultaneous detection of anti-*T*. *cruzi* IgG1 reactivity to distinct target antigens (amastigote-“A”, trypomastigote-“T” and epimastigote-“E”) from TcI, TcVI and TcII DTUs. The ability of Chagas-Flow ATE IgG1 to accomplish the genotype-specific serodiagnosis is based on the use of specific sets of target antigens to accomplish the genotype-specific sero-classification of Chagas disease patients [24].

In the present study, the Chagas-Flow ATE IgG1 methodology was used for post-therapeutic monitoring of chronic Chagas disease patients, aiming at identifying changes in *T*. *cruzi* genotype-specific serological profile upon Benznidazole etiological treatment. This approach is relevant to provide novel insights to support the relevance of accomplishing the genotype-specific serodiagnosis during clinical post-therapeutic management of chronic Chagas disease patients.

## Methods

### Ethics statement

The study was submitted and approved by Ethics Committees at Instituto René Rachou-FIOCRUZ-Minas (C.A.A.E: 26890014.6.0000.5091, protocol number #3.055.734) and Universidade Federal de Ouro Preto (C.A.A.E: 26890014.6.3001.5150, protocol number # 766.573). All participants have read and sign the informed consent form before starting the study. All the experiments were performed in accordance with relevant guidelines and regulations.

### Study population

The present investigation included a non-probabilistic convenience sampling from archival biorepository maintained at Grupo Integrado de Pesquisas em Biomarcadores/Instituto René Rachou- FIOCRUZ-Minas. The study population comprised a total 46 patients with chronic Chagas disease (CH) enrolled at two time points (Baseline and 5^Years^ Follow-up) and a control group composed of eight Non-Infected subjects (NI).

The CH group included Chagas disease patients of both sexes (15 males and 31 females), age ranging from 21 to 60 years old, residents of distinct Chagas disease endemic municipalities from Minas Gerais State, Brazil. Although the precise mechanism of infection is unknown, the *T*. *cruzi* infection was acquired congenitally or mostly by vectorial transmission at early childhood. Therefore, all patients included in the CH group were at the chronic phase of Chagas disease. The patients were invited to participate in the study during to routine medical appointment at the Ambulatory of Chagas Disease from Hospital das Clínicas, Universidade Federal de Minas Gerais, from 1995 to 2005. Chagas disease patients were classified into two subgroups, referred as: Not Treated (NT, n = 16; 4 males and 12 females; mean age = 38 years old) and Benznidazole Treated (Bz-T, n = 30; 11 males and 19 females; mean age = 36 years old). The Benznidazole Treated group was composed of patients who received the standard Chagas disease treatment according to the guidelines from the Brazilian Health Ministry, consisting of 5mg/kg/day for 60 consecutive days. The Not Treated group comprised patients that refused to receive the standard Chagas disease treatment but agreed to participate in the study. All patients remained under continuous medical supervision and assistance.

The NI group included subjects of both sexes (2 males and 6 females), age ranging from 27 to 45 years old, residents of distinct municipalities from Minas Gerais State, Brazil.

**Fig 1:**
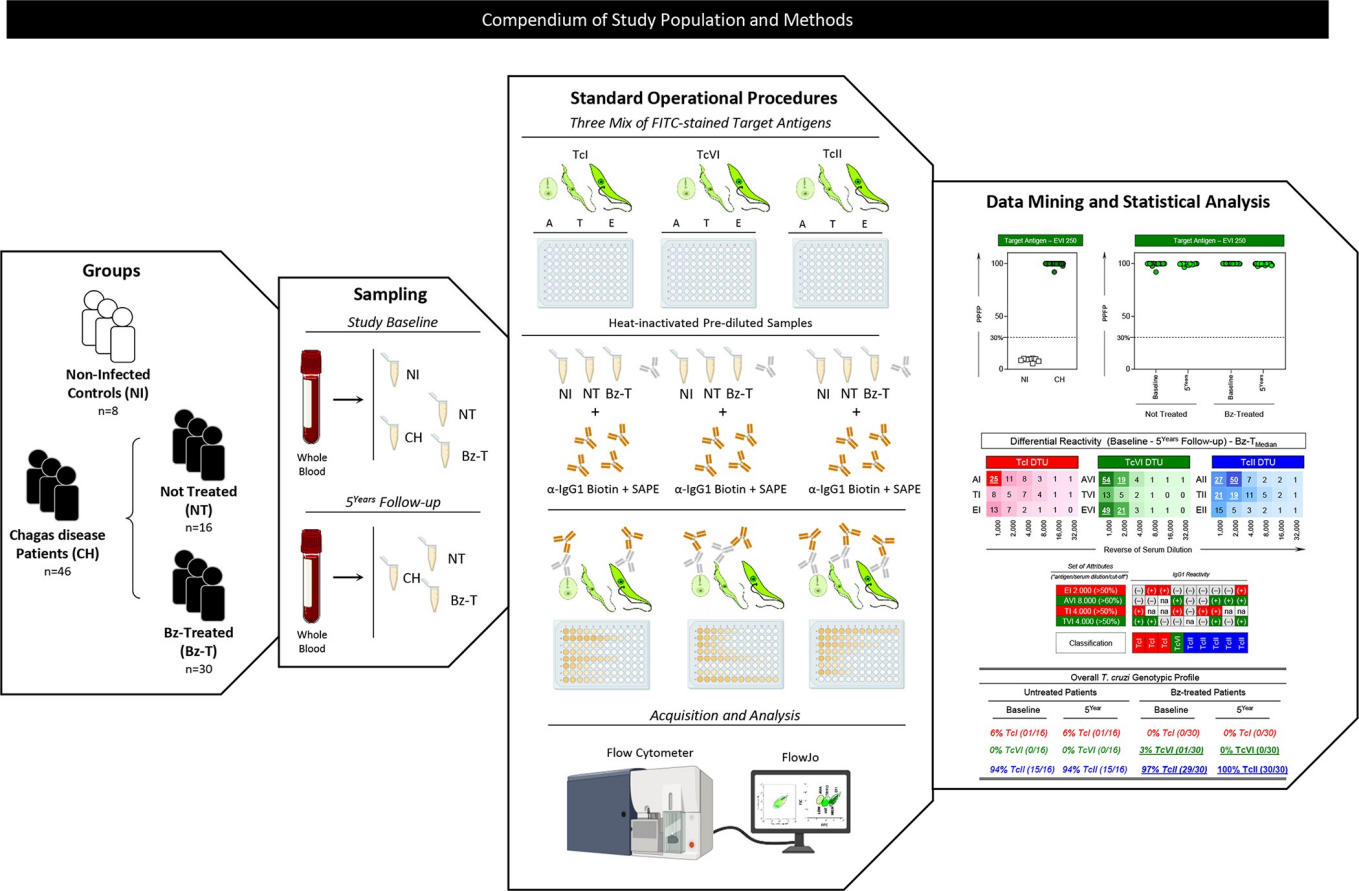
Compendium of study population and methods. An overview of study groups, sampling, standard procedures, data mining and statistical analysis summarizes the compendium of study population and methods. The study groups comprised a non-probabilistic convenience sampling from archival biorepository, including 54 adult subjects, both sexes, referred as: chronic Chagas disease patients (CH, n = 46) and non-infected healthy subjects (NI, n = 8). CH were further classified into two subgroups, named: Not Treated (NT, n = 16) and Benznidazole Treated (Bz-T, n = 30). Serum specimens from CH were collected at two time points: at Study Baseline and at 5^Years^ Follow-up. Chagas-Flow ATE IgG1 standard procedure was carried out as previously reported by Alessio *et al*. (2020) [24]. FITC-labeled parasites (ATE target antigen mix) were incubated with heat-inactivated pre-diluted samples followed by addition of second step reagents (biotin-conjugated anti-human IgG1 antibody plus streptavidin phycoerytrin–SAPE). TcI, TcVI or TcII Chagas-Flow ATE IgG1 were performed in simultaneous assays. Parasite suspensions were acquired in a FACSCalibur flow cytometer (BD Bioscience, San Diego, CA, USA). Distinct approaches were used for data mining and statistical analysis, including: IgG1 reactivity, expressed as percentage of positive fluorescent parasites (PPFP) to specific target antigens; differential median reactivity (Study Baseline– 5^Years^ Follow-up) of CH subgroups (NT and Bz-T) and changes in genotype-specific serological profiles upon Bz-treatment were assessed using specific sets of TcI, TcVI and TcII target antigens reported in reactivity boards.

Serum specimens from CH were collected at two time points: at Study Baseline and at 5^Years^ Follow-up. Samples from NI were obtained at a single time point at enrollment. A total of 100 serum samples (NI, n = 8; NT, n = 32 and Bz-T, n = 60) were tested. Serum aliquots stored at -80°C were heat-inactivated (56°C for 30 min) prior use for Chagas-Flow ATE IgG1. **Fig 1** summarizes the compendium of the study population and sampling.

### *T*. *cruzi* target antigens

The *T*. *cruzi* target antigens used in the Chagas-Flow ATE IgG1 methodology comprises of three *Discrete Type Units*, including: TcI DTU (Colombian strain), TcVI DTU (CL strain) and TcII DTU (Y strain). The *T*. *cruzi* strains were obtained from the cryobank maintained at Grupo Integrado de Pesquisas em Biomarcadores, Instituto René Rachou, FIOCRUZ-Minas.

The *T*. *cruzi* evolutive forms (amastigote-“A”, trypomastigote-“T” and epimastigote-“E”) from TcI, TcVI and TcII DTUs were obtained as previously described by to Alessio *et al*. (2014) [27]. Briefly, alive “A” and “T” forms, harvested from L929 cell line cultures were labeled with fluorescein isothiocyanate (FITC) at 37°C for 30 min and maintained at 37°C for 60 min to accomplish the differential FITC-staining, according to Alessio *et al*. (2014) [27]. “E” forms obtained from axenic *in vitro* culture in “Liver Infusion Tryptose” (LIT) medium [30] were paraformaldehyde-fixed overnight, labeled with FITC at 37°C for 30 min and maintained overnight at 4°C to stabilize the FITC-staining. Three distinct target antigen mix, referred as: TcI, TcVI and TcII were prepared to obtain equivalent proportions of FITC-labeled of *T*. *cruzi* evolutive forms (33% “A”, 33% “T” and 33% “E”). The FITC-staining profile of *T*. *cruzi* evolutive forms were monitored by flow cytometry before each experimental batch, as a quality control recommended for good laboratory practice. The FITC-stained target antigen mix of TcI, TcVI and TcII DTU were individually run in simultaneous assays.

### Chagas-Flow ATE IgG1 standard procedure

Chagas-Flow ATE IgG1 was carried out as previously reported by Alessio *et al*. (2020) [24]. Briefly, in U-bottom 96-well plates, 50μL aliquots of pre-diluted serum samples (1:1,000 to 1:32,000) were incubated with 50μL of the ATE target antigen mix (TcI, TcVI or TcII DTU in simultaneous assays) at 37°C for 30 min. Following, parasites were washing twice and incubated with 50μL of biotin-conjugated anti-human IgG1 antibody (1:6,400) together with 20μL of streptavidin phycoerytrin–SAPE (1:400) at 37°C for 30 min. After two washing steps, the parasite suspension fixed and stored at 4°C prior acquisition of 10,000 events/sample in a FACSCalibur flow cytometer (BD Bioscience, San Diego, CA, USA). Positive and negative control samples as well as second step reagents monitoring were included on each experimental assay. The FlowJo software Version 10.1 (BD Biosciences, San Diego, CA, USA) were used for data analyses. The IgG1 reactivity to each target antigen (“A”, “T” and “E” from TcI, TcVI or TcII DTU) was expressed as percentage of positive fluorescent parasites (PPFP) determined over the positivity limit of PPFP<2% set for the second step reagent internal control, according to Alessio *et al*. (2014) [27]. **Fig 1** summarizes the major steps of the standard operational procedure. The detailed description of the criteria used to define the cut-offs employed for each target antigen were previously described by Alessio *et al*. (2020) [24].

### Data analysis

Descriptive statistics were used to characterize the overall IgG1 reactivity profile of CH samples to distinct TcI, TcVI and TcII target antigens, at Study Baseline and 5^Years^ Follow-up. Differential median reactivity (Study Baseline– 5^Years^ Follow-up) was assessed for CH subgroups (NT and Bz-T). Comparative analysis between PPFP median values observed at Study Baseline and 5^Years^ Follow-up was carried out by Wilcoxon test and significance considered at *p <0,05, **p<0,001, ***p<0,0001, ****p<0,00001. Changes in genotype-specific serological profiles were assessed using specific sets of TcI, TcVI and TcII target antigens reported in reactivity boards. The GraphPad Prism software, Version 5.0 (San Diego, CA, USA) was used for statistical analysis and graphical arts. Microsoft Excel 2010 was used to construct reactivity boards and graphical arts. **Fig 1** summarizes the strategies used for data mining and statistical analysis.

## Results

### Anti-*T*. *cruzi* antibody reactivity of serum samples from Chagas disease patients at study baseline using EVI target antigen for universal diagnosis purpose

**Fig 2:**
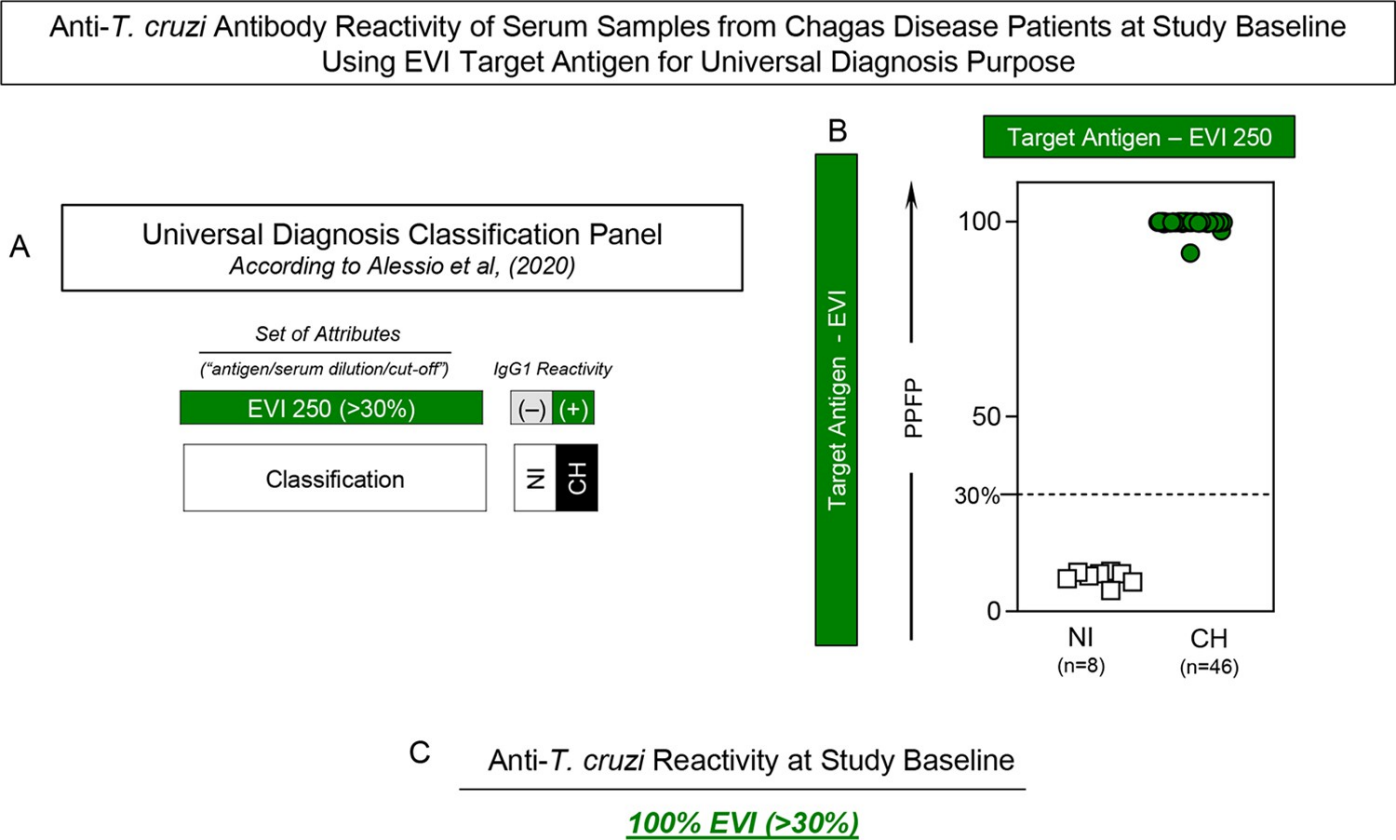
Anti-*T*. *cruzi* antibody reactivity of serum samples from Chagas disease patients at study baseline using EVI target antigen for universal diagnosis purpose. (A) Classification panel showing the set of attributes (“antigen/serum dilution/cut-off”) employed for the universal diagnosis of Chagas disease by Chagas-Flow ATE IgG1, according to Alessio *et al*. (2020) [24]. The set of attributes “EVI 250/30%” were used in this study to classify the serum samples from Chagas disease patients (CH, n = 46) from non-infected healthy subjects (NI, n = 8). (B) Anti-EVI IgG1 reactivity of serum samples (1:250 dilution) from CH at Study Baseline (green dots) *vs* NI (white rectangles). The results are presented in scatter plot distribution of individual values expressed as percentage of positive fluorescent parasites (PPFP). The dotted line represents the PPFP cut-off (30%) used to classify the serum samples. (C) Overall anti-*T*. *cruzi* reactivity profile at Study Baseline.

The analysis of anti-EVI IgG1 reactivity by Chagas-Flow ATE IgG1 has been proposed by Alessio *et al*. (2020) [24] as a classification panel to accomplish the universal diagnosis of Chagas disease. In this line, these set of attributes (“antigens/serum dilution/cut-off”) was assessed in serum samples from Chagas disease patients and non-infected healthy subjects at Study Baseline and the results are shown in **Fig 2**. The classification panel previously proposed by Alessio *et al*. (2020) [24] for universal diagnosis of Chagas disease by Chagas-Flow ATE IgG1 is presented in **Fig 2A**. Based on this criterion, all serum samples from CH presented positive results, confirming the diagnosis of Chagas disease at Study Baseline. Conversely, all samples from NI exhibited negative results, re-emphasizing the specificity of Chagas-Flow ATE IgG1 (**Fig 2B**). Overall, the anti-*T*. *cruzi* reactivity profile showed 100% of seropositivity in CH at Study Baseline (**Fig 2C**).

### Anti-*T*. *cruzi* antibody reactivity of serum samples from Chagas disease patients at study baseline using distinct target antigens employed for genotype-specific serodiagnosis purpose

**Fig 3:**
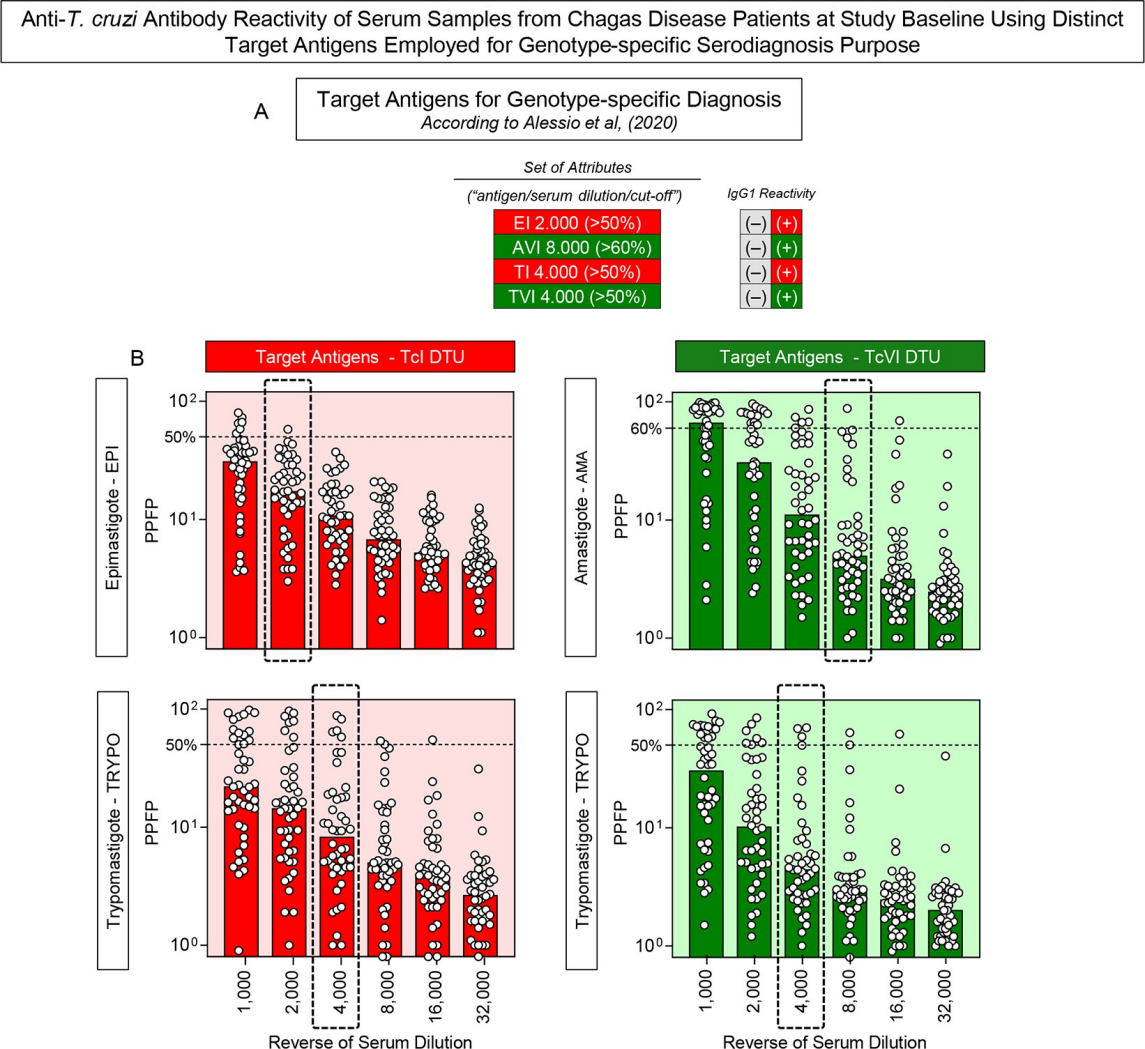
Anti-*T*. *cruzi* antibody reactivity of serum samples from Chagas disease patients at study baseline using distinct target antigens employed for genotype-specific serodiagnosis purpose. (A) Summary of attribute sets (“antigens/serum dilutions/cut-offs”) used for genotype-specific serodiagnosis of Chagas disease, according to Alessio *et al*. (2020) [24]. (B) IgG1 reactivity profile of serum samples from Chagas disease patients at Study Baseline (n = 46) to distinct target antigens: epimastigote (EPI) and trypomastigote (TRYPO) from TcI and amastigote (AMA) and trypomastigote (TRYPO) from TcVI *T*. *cruzi* DTUs along the titration curve (1:1,000 to 1:32,000). The results are presented in scatter plot distribution of individual values over bars (median) expressed as the percentage of positive fluorescent parasites (PPFP). The set of attributes used for genotype-specific serology of Chagas disease by Chagas-Flow ATE IgG1 are underscored, comprising: “target antigens”, “serum dilution” (dashed rectangles) and “cut-off” (dashed lines).

Aiming at performing the genotype-specific serological diagnosis of the Chagas disease patients enrolled in the present investigation, the IgG1 reactivity profile of serum samples from CH was characterize at Study Baseline and the results presented in **Fig 3**. Alessio *et al*. (2020) [24] have proposed the use of a set of attributes to accomplish the genotype-specific sero-classification of Chagas disease patients, comprising: “EI 2,000/50%”; “AVI 8,000/60%”; “TI 4,000/50%” and “TVI 4,000/50%” (**Fig 3A**). These attributes were highlighted along the titration curve (1:1,000 to 1:32,000), to subsidize the genotype-specific serological diagnosis of the Chagas disease patients at Study Baseline (**Fig 3B**).

**Fig 4:**
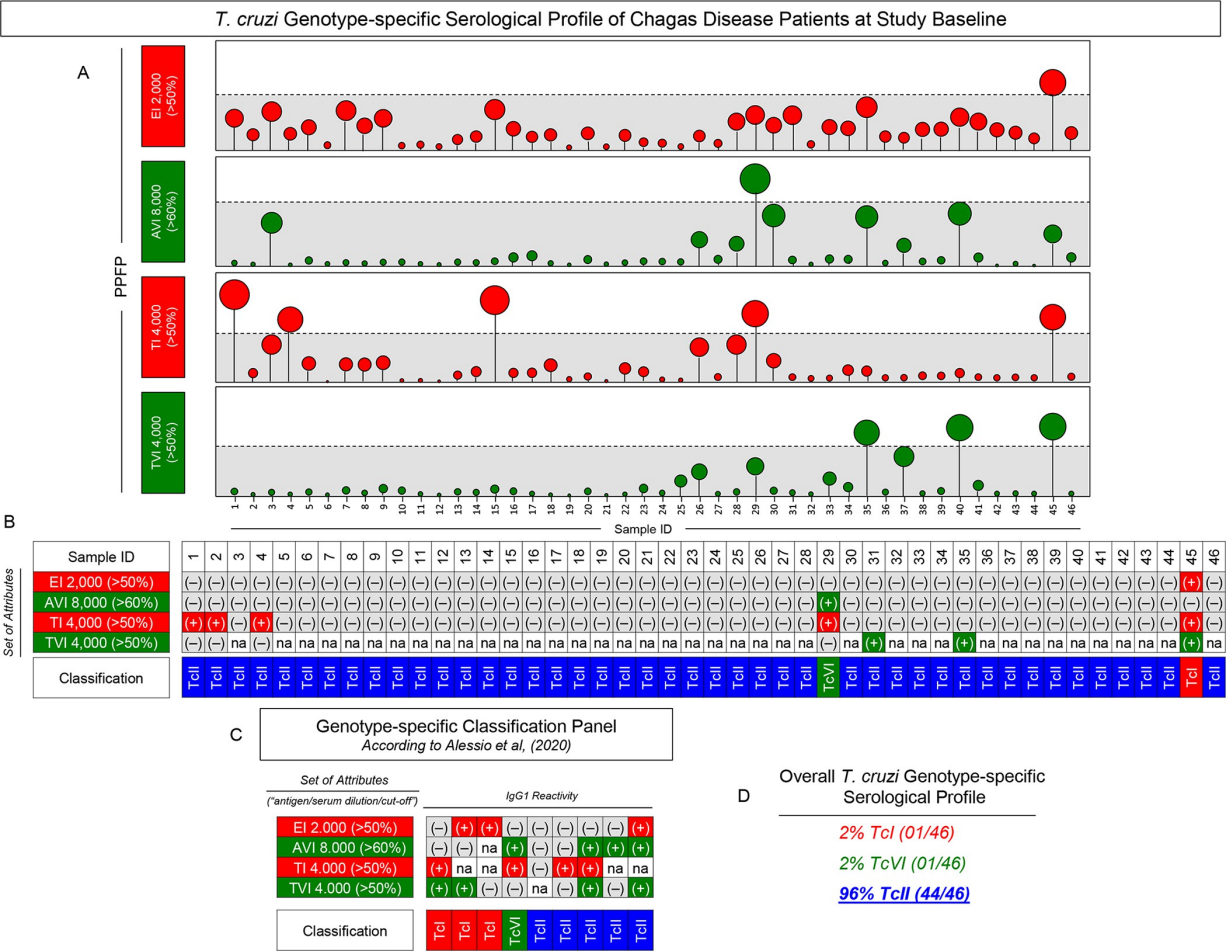
*T*. *cruzi* genotype-specific serological profile of Chagas disease patients at study baseline. (A) The IgG1 reactivity profile of serum samples from Chagas disease patients (n = 46) at Study Baseline determined by Chagas-Flow ATE IgG1, using the selected sets of attributes: EI 2,000 (>50%), AVI 8,000 (>60%), TI 4,000 (>50%) and TVI 4,000 (>50%). The results are presented in lollypop charts of individual values expressed as the percentage of positive fluorescent parasites (PPFP). Samples with positive results using each set of attributes were identified outside the gray background zone established according to the cut-off edges. (B) Reactivity dashed board using the selected set of attributes to define the genotype-specific sero-classification of individual samples. (C) Classification panel showing the set of attributes (“antigen/serum dilution/cut-off”) employed for the genotype-specific serodiagnosis of Chagas disease by Chagas-Flow ATE IgG1, according to Alessio *et al*. (2020) [24]. Color keys illustrate the genotype-specific serological classification as TcI (red), TcVI (green), TcII (blue) DTUs based on positive (+) or negative (-) reactivity with distinct target antigens. na = not applicable. (D) Overall *T*. *cruzi* genotype-specific serological profile of Chagas disease patients (n = 46) at Study Baseline.

Using these attributes, the genotype-specific serodiagnosis of Chagas disease patients was accomplished at Study Baseline and the results presented in **Fig 4**. Based on the overall profile of Chagas-Flow ATE IgG1 reactivity (**Fig 4A**), a reactivity board was assembled to classify the individual samples of Chagas disease patients (**Fig 4B**). Using the criteria of genotype-specific serology proposed by Alessio *et al*. (2020) [24] (**Fig 4C**), data demonstrated that, at Study Baseline, 96% of the Chagas disease patients (44/46) presented a serological profile compatible with TcII genotype infection. One patient was classified as infected by TcI DTU and one identified as infected by TcVI DTU (**Fig 4D**).

### Impact of Bz-treatment on anti-*T*. *cruzi* antibody reactivity of serum samples from Chagas disease patients using EVI target antigen for monitoring purpose (Study Baseline vs 5^Years^ Follow-up)

**Fig 5:**
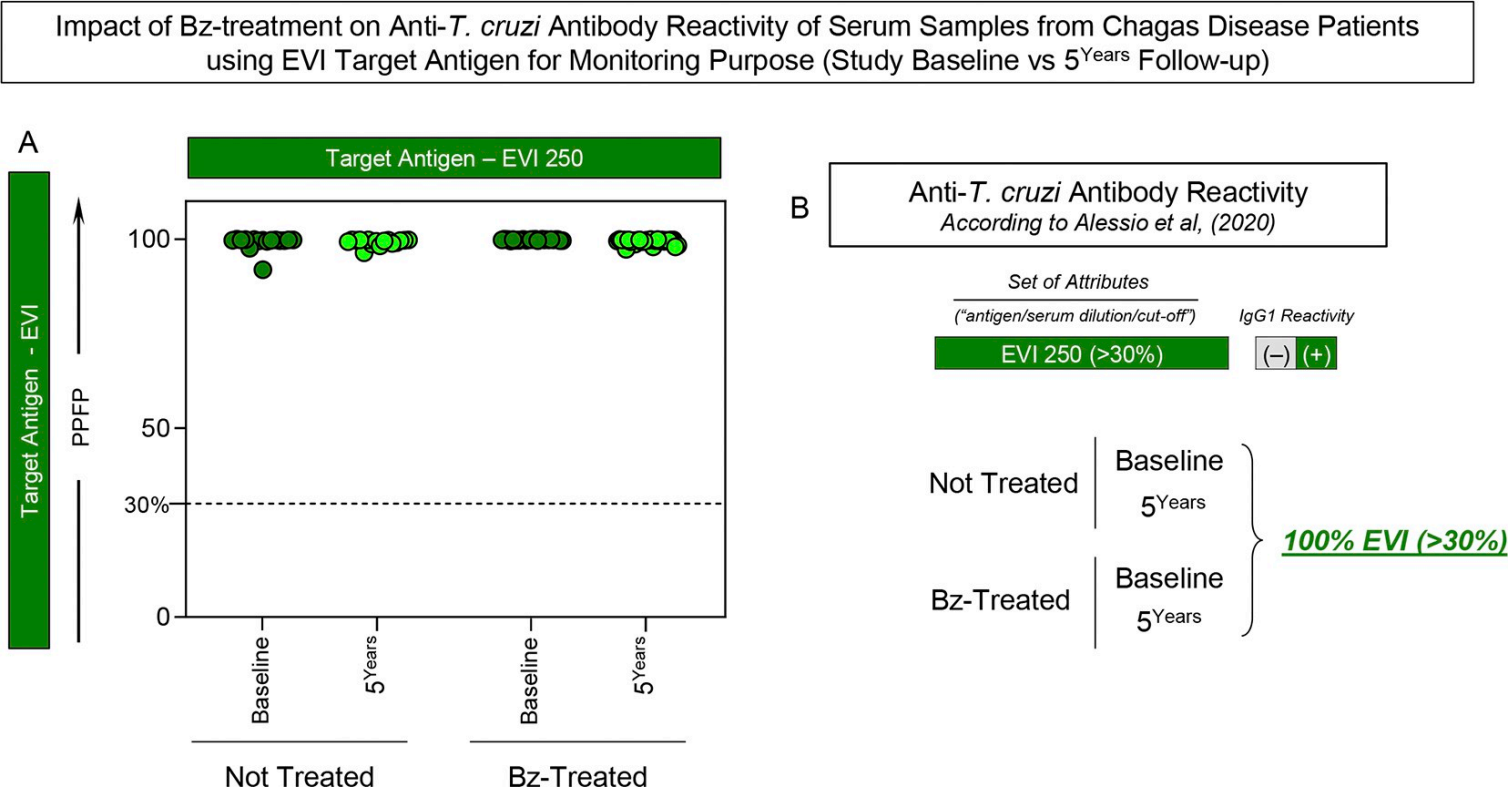
Impact of Bz-treatment on anti-*T*. *cruzi* antibody reactivity of serum samples from Chagas disease patients using EVI target antigen for monitoring purpose (Study Baseline vs 5^Years^ Follow-up). (A) IgG1 reactivity of Not Treated (NT, n = 16) and Benznidazole Treated (Bz-T, n = 30) Chagas disease patients at Study Baseline (dark green) and at 5^Years^ Follow-up (light green) using the set of attributes “EVI 250/30%” as proposed by Alessio *et al*. (2020) [24]. The results are presented in scatter plot of individual values expressed as the percentage of positive fluorescent parasites (PPFP) with the cut-off represented by the dotted line. (B) Classification panel showing the set of attributes (“antigen/serum dilution/cut-off”) employed for the monitoring of Chagas disease by Chagas-Flow ATE IgG1, according to Alessio *et al*. (2020) [24]. Overall anti-*T*. *cruzi* reactivity profile at Study Baseline and at 5^Years^ Follow-up.

Aiming at investigating whether the anti-EVI IgG1 reactivity was impacted by the Bz-treatment, the set of attributes “EVI 250/30%” was assessed by Chagas-Flow ATE IgG1 in serum samples from Not Treated (NT) and Benznidazole Treated (Bz-T) Chagas disease patients at Study Baseline and 5^Years^ Follow-up and the results are shown in **Fig 5**. In this line, the post-therapeutic monitoring was performed using the criterion proposed by Alessio *et al*. (2020) [24]. The results demonstrated that the anti-EVI IgG1 reactivity of serum samples from NT did not differ at 5^Years^ Follow-up as compared to Study Baseline. Likewise, all serum samples from Bz-T presented similar anti-EVI IgG1 reactivity at both timepoints, demonstrating that no serological changes occurred within the 5 years monitoring upon Bz-treatment (**Fig 5B**).

### Impact of Bz-treatment on anti-*T*. *cruzi* antibody reactivity of serum samples from Chagas disease patients using distinct target antigens employed for genotype-specific serodiagnosis purpose (Study Baseline vs 5^Years^ Follow-up)

**Fig 6:**
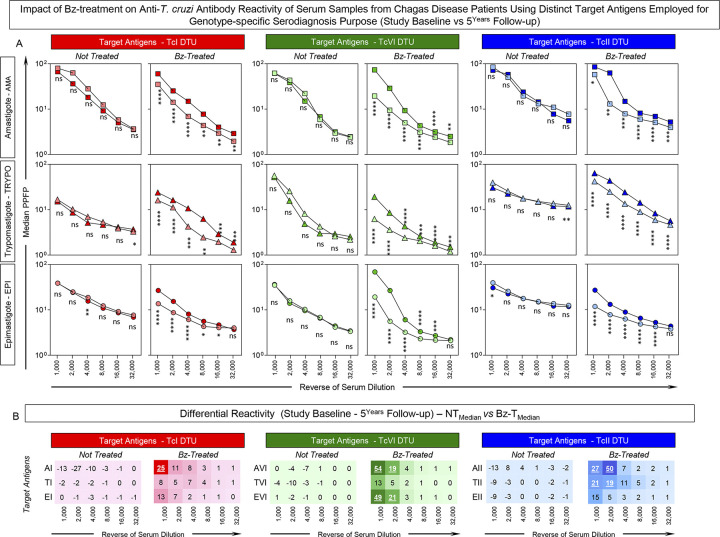
Impact of Bz-treatment on anti-*T*. *cruzi* antibody reactivity of serum samples from Chagas disease patients using distinct target antigens employed for genotype-specific serodiagnosis purpose (Study Baseline vs 5^Years^ Follow-up). (A) Anti-*T*. *cruzi* IgG1 reactivity of serum samples from Not Treated (NT, n = 16) and Benznidazole Treated (Bz-T, n = 30) Chagas disease patients at Study Baseline (dark symbols) and at 5^Years^ Follow-up (light symbols) with distinct target antigens: amastigote (AMA), trypomastigote (TRYPO) and epimastigote (EPI) from TcI, TcVI and TcII *T*. *cruzi* DTUs along the titration curve (1:1,000 to 1:32,000). The results are presented in line charts of median values expressed as percentage of positive fluorescent parasites (PPFP). Comparative analyses were carried out by Wilcoxon test and significance considered at *p <0,05, **p<0,001, ***p<0,0001, ****p<0,00001. ns = no significant difference. (B) Differential median reactivity (Study Baseline– 5^Years^ Follow-up) was assessed for NT and Bz-T considering distinct target antigens along the titration curve. The target antigens and serum dilutions presenting highest differential reactivity are underscored by bold underline format.

The genotype-specific IgG1 reactivity to distinct target antigens was characterized in paired serum samples from Not Treated (NT) and Benznidazole Treated (Bz-T) Chagas disease patients at 5^Years^ Follow-up and the results presented in **Fig 6**. Overall, no changes in the median IgG1 reactivity were observed for serum samples from NT tested along the titration curve (1:1,000 to 1:32,000) at 5^Years^ Follow-up as compared to Study Baseline (**Fig 6A**). On the other hand, significant differences in the IgG1 reactivity were detected for Bz-T in most serum dilutions tested at 5^Years^ Follow-up as compared to Study Baseline (**Fig 6A**).

The differential IgG1 reactivity (Study Baseline– 5^Years^ Follow-up) was further calculated for serum samples from NT and Bz-T and the results presented in **Fig 6B**. Data demonstrated that minor differences were observed for NT, characterized by null or low negative values, while major changes were found for Bz-T. The most outstanding shifts identified for Bz-T, were observed in the sets “AVI 1,000”, “AII 2,000” and “EVI 1,000” (54%, 50% and 49%, respectively) (**Fig 6B**).

### Impact of Bz-treatment on *T*. *cruzi* genotype-specific serological profile of samples from Chagas disease patients (Study Baseline vs 5^Years^ Follow-up)

**Fig 7:**
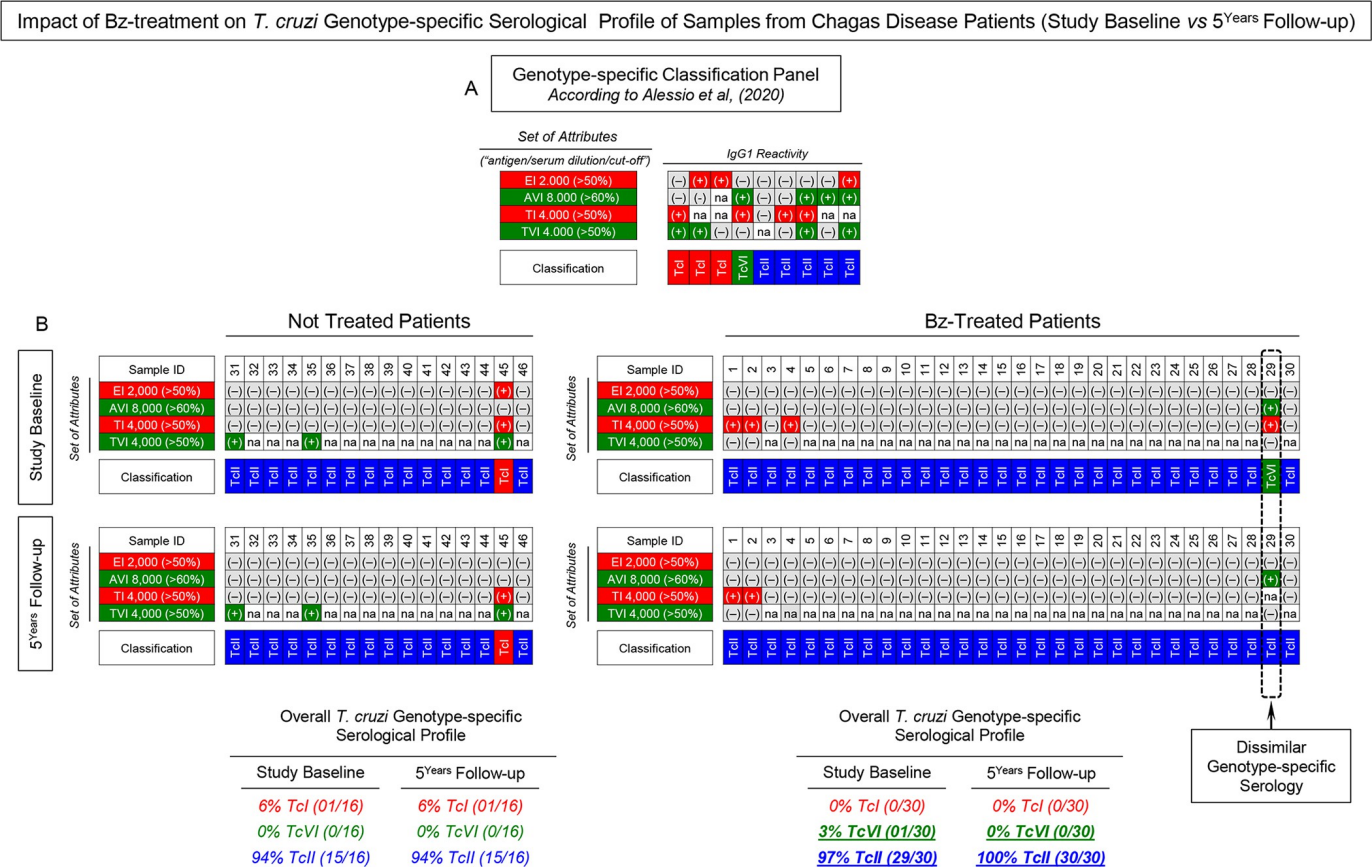
Impact of Bz-treatment on *T*. *cruzi* genotype-specific serological profile of samples from Chagas disease patients (Study Baseline vs 5^Years^ Follow-up). (A) Classification panel showing the set of attributes (“antigen/serum dilution/cut-off”) employed for the genotype-specific serodiagnosis of Chagas disease by Chagas-Flow ATE IgG1, according to Alessio *et al*. (2020) [24]. Color keys illustrate the genotype-specific sero-classification as TcI (red), TcVI (green), TcII (blue) DTUs based on positive (+) or negative (-) reactivity to distinct target antigens. na = not applicable. (B) Overall *T*. *cruzi* genotype-specific serological profile of Not Treated (NT, n = 16) and Benznidazole Treated (Bz-T, n = 30) Chagas disease patients at Study Baseline and at 5^Years^ Follow-up. Changes in the *T*. *cruzi* genotype-specific serology at 5^Years^ Follow-up as compared to Study Baseline are underscored by dotted rectangle.

Intending to verify whether changes in the *T*. *cruzi* genotype-specific serology would occur at 5^Years^ Follow-up as compared to Study Baseline, the genotype-specific serological profile of NT and Bz-T was characterized, and the results presented in **Fig 7**. The general criteria proposed by Alessio *et al*. (2020) [24] was employed for genotype-specific serodiagnosis (**Fig 7A**). Using the criteria, a reactivity board was constructed to classify NT and Bz-T individual samples at both timepoints (**Fig 7B).** Data demonstrated that no changes were observed for the genotype-specific IgG1 reactivity of NT samples tested at Study Baseline and at 5^Years^ Follow-up (TcI = 6%; TcVI = 0%; TcII = 94%). Conversely, dissimilar *T*. *cruzi* genotype-specific serology was detected in Bz-T samples tested at Study Baseline (TcI = 0%; TcVI = 3%; TcII = 97%) as compared to those tested at 5^Years^ Follow-up (TcI = 0%; TcVI = 0%; TcII = 100%).

**Fig 8:**
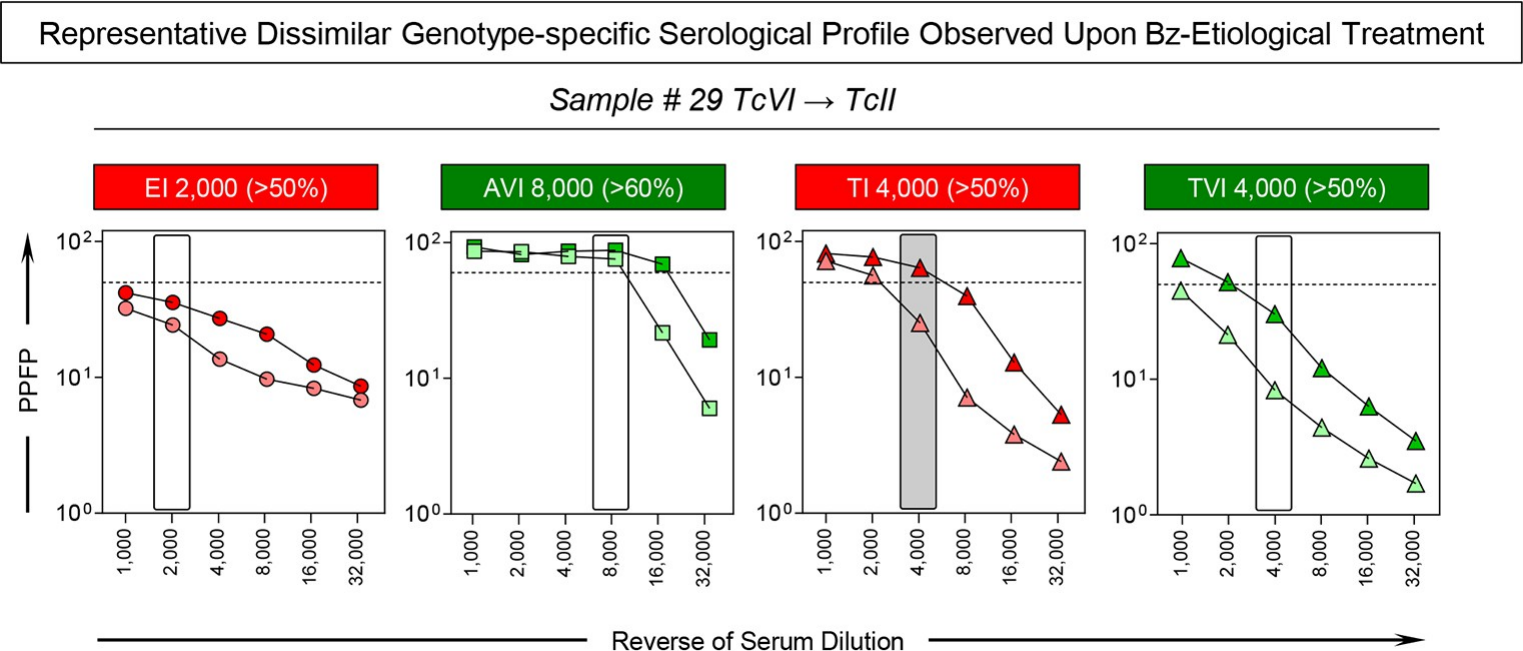
Representative dissimilar genotype-specific serological profile observed upon Bz-etiological treatment. Anti-*T*. *cruzi* IgG1 reactivity of one serum sample from Benznidazole Treated Chagas disease patient (#29) presenting dissimilar *T*. *cruzi* genotype-specific serological profile at 5^Years^ Follow-up (light symbols) as compared to Study Baseline (dark symbols), using the sets of attributes (rectangles) proposed by Alessio *et al*. (2020) [24] for genotype-specific serodiagnosis: EI 2,000 (>50%), AVI 8,000 (>60%), TI 4,000 (>50%) and TVI 4,000 (>50%). The results are presented in line charts of individual values expressed as percentage of positive fluorescent parasites (PPFP) with the cut-off represented by the dotted line. The dark rectangle underscores the set of attributes “TI 4,000 (>50%)” exhibiting the dissimilar IgG1 reactivity profile at 5^Years^ Follow-up as compared to Study Baseline.

The dissimilar particularly genotype-specific serological profile was observed for the patient #29, who was classified as infected with TcVI DTU at Study Baseline and as TcII DTU at 5^Years^ Follow-up (**Fig 7B, dashed rectangle**). Representative profile of changes of genotype-specific IgG1 reactivity observed for the patient #29 before and after Benznidazole etiological treatment is shown in **Fig 8**. Overlaid profiles along the titration curves (1:1,000 to 1:32,000) were assembled to compare the IgG1 reactivity for the set of attributes “EI 2,000/50%, “AVI 8,000/60%”, “TI 4,000/50%” and “TVI 4,000/50%” at Study Baseline and at 5^Years^ Follow-up. The shift of the IgG1 reactivity, encompassing the set “TI 4,000/50%” (PPFP > 50% towards PPFP < 50%), was the parameter accountable for the change genotype-specific sero-classification reported for the patient #29. The IgG1 reactivity for the sets “EI 2,000/50%, “AVI 8,000/60%” and “TVI 4,000/50%” remained unaltered across the cut-off edges (**Fig 8**).

## Discussion

The present study aimed to verify the impact of etiological treatment on the genotype-specific serodiagnosis of chronic Chagas disease patients. For this purpose, the Chagas-Flow ATE IgG1 methodology, originally described by Alessio *et al*. (2020) [24], was employed to characterize, at Study Baseline and 5^Years^ Follow-up, the reactivity profile of serum samples from Chagas disease patients categorized as Not Treated and Benznidazole-Treated. The Chagas-Flow ATE IgG1 is a competitive platform that simultaneously use the “A”, “T” and “E” evolutive forms to achieve the selective binding high affinity IgG1 to each target antigen. As proposed by Alessio *et al*. (2020) [24], distinct set of attributes (“antigen/serum dilution/cut-off”) are required to accomplish the universal and genotype-specific serodiagnosis of Chagas disease. In this line, the use of antigens other than the selected targets is relevant to minimize the cross reactivity that may interfere in the specificity of Chagas-Flow ATE IgG1.

At Study Baseline, all serum samples from Chagas disease patients presented positive results in the Chagas-Flow ATE IgG1, according to the reactivity profile with the set of attributes “EVI 250/30%”. These findings corroborate the previous reports from Alessio *et al*. (2020) [24], demonstrating that this set of attributes exhibit enhanced performance to accomplish the universal diagnosis of Chagas disease. The genotype-specific serodiagnosis further demonstrated that at Study Baseline most serum samples presented a reactivity profile compatible with the infection with TcII DTU, except for one sample from NT (TcI) and another from Bz-T (TcVI) subgroups. The predominance of TcII DTU amongst Chagas disease patients enrolled in the present investigation reflect the high prevalence of TcII *T*. *cruzi* genotype in the domestic cycle of transmission in Brazil [5,31,32].

Previous studies have postulated that *T*. *cruzi* genetic variability is closely related to distinct parasite biology features and may lead to the development different clinical aspects [5,17,31,33,34]. Moreover, it has been previously reported that *T*. *cruzi* genetic variability is also associated with the effectiveness therapeutic response of Chagas disease [13,15,17,31,33–38]. In this sense, previous studies have demonstrated that hosts infected with distinct *T*. *cruzi* genotypes exhibited differential susceptibility to etiological treatment [12,39–42]. Clones and strains belonging to the TcI *T*. *cruzi* DTU presented higher resistance to Benznidazole treatment as compared to the TcII and TcVI DTUs [12,39–43]. Thus, it is important evaluated the impact of Benznidazole treatment in the *T*. *cruzi* genetic.

At 5^Years^ Follow-up, all serum samples from Chagas disease patients, both NT and Bz-T, remained with positive reactivity in the Chagas-Flow ATE IgG1, according to the results obtained with the set of attributes “EVI 250/30%”. The anti-EVI IgG1 reactivity has been proposed by Alessio *et al*. (2014) [27] for post-therapeutic monitoring of Chagas disease, showing outstanding ability to discriminate NT from treated not-cured and treated cured patients following Bz-T. In the present study, the use of the attributes (EVI 250) and the 30% cut-off demonstrated that all Bz-T patients remained with positive reactivity at 5^Years^ Follow-up, suggesting therapeutic failure. However, previous studies have suggested that monitoring of the Bz-therapeutic efficacy of patients treated during chronic phase of Chagas disease may require a follow-up time over 10 years [2,4,11]. Therefore, the therapeutic failure observed for all Bz-treated patients enrolled in the present investigation may reflect the timespan elapsed since Bz-treatment and the 5^Years^ Follow-up. Additionally, it is important to mention that Bz-therapeutic response differs considerable amongst distinct *T*. *cruzi* genotypes [5,44]. Considering our findings that 29 out 30 Bz-treated patients (97%) were infected with TcII, a well-known Bz-partially resistant *T*. *cruzi* genotype, it is likely to expect that therapeutic failure may occurred in most Bz-treated patients.

Previous studies have demonstrated that changes in serological reactivity to distinct *T*. *cruzi* antigens may occur in Bz-treated Chagas disease patients even when the anti-epimastigote IgG reactivity remain unaltered [20]. Intended to verify putative changes in the overall IgG1 reactivity to TcI, TcVI and TcII target antigens may occur from Baseline towards 5^Years^ Follow-up, paired serum samples from Not Treated (NT) and Benznidazole Treated (Bz-T) Chagas disease patients were tested along the titration curve. In this sense, it is worth mentioning that regardless all samples from Bz-T remained with positive at 5^Years^ Follow-up using the set of attributes “EVI 250/30%”, the median reactivity detected to other sets of attributes (“AVI 1,000”, “AII 2,000” and “EVI 1,000”) displayed lower reactivity profile.

These putative changes in the overall IgG1 reactivity could impact the genotype-specific serology. However, the changes of IgG1 reactivity observed did not impact the genotype-specific serodiagnosis of most Bz-treated Chagas disease patients (29/30) that remained with the same genotype-specific serological profile Baseline towards 5^Years^ Follow-up. Of note, one out of 30 Bz-T samples (sample #29) exhibited a dissimilar *T*. *cruzi* genotype-specific serology, being classified as TcVI at Study Baseline and as TcII at 5^Years^ Follow-up. The post-treatment “TI 4,000/50%” signature was dissimilar to the pre-treatment counterpart (PPFP > 50% towards PPFP < 50%) in the same patient, suggesting that this attribute is more sensitive to the selection of *T*. *cruzi* DTU between Study Baseline and 5^Years^ Follow-up. We hypothesized that the patient #29 may presented a mixed *T*. *cruzi* infection (TcVI + TcII) and that upon Bz-etiological treatment, the TcVI (Bz-susceptible) was possibly eliminated and the TcII (Bz-resistant) persists as the refractory population due to treatment selection. It is unlikely that patient #29 was reinfected by *T*. *cruzi* between Bz-treatment and the 5^Years^ Follow-up, considering the control of *T*. *cruzi* vectorial transmission in Brazil, according to the Pan American Health Organization 2006 milestone, conferring to the Brazilian Ministry of Health the international certificate of Chagas disease transmission elimination. This achievement has been confirmed by an international expert commission based on visits to all Brazilian States [45]. Previous works demonstrated that TcVI DTU are more susceptible to treatment than TcII DTU of *T*. *cruzi* [12,42,43,46]. Wild populations of *T*. *cruzi* may comprise both susceptible and resistant DTUs to Benznidazole treatment, therefore destruction of susceptible forms leads to the selection and proliferation of resistant subpopulations, as a consequence of drug-driven selective pressure [47,48]. Natural variations of drug susceptibility between *T*. *cruzi* strains are supposed to be one of the most important factors that explaining the low rates of cure in some treated chronic Chagas disease patients [12,40,49–52].

The present study has some limitations. This is a single-center study with the small sample size. Additional multicentric investigations with a larger number of patients from distinct geographical areas of infections with distinct *T*. *cruzi* genotypes would provide more accurate data to evaluate the impact of therapeutic intervention inducing changes in *T*. *cruzi* genotype-specific serological profile in Bz-treated Chagas disease patients.

In conclusion, our findings of dissimilar *T*. *cruzi* DTU profile detected upon Bz-treatment re-emphasize the relevance of accomplishing the genotype-specific serodiagnosis during clinical post-therapeutic management of chronic Chagas disease patients.
